# Nano Reviews in its 4th Volume

**DOI:** 10.3402/nano.v4i0.21593

**Published:** 2013-06-11

**Authors:** Vasudevanpillai Biju

*Nano Reviews* – now in its 4th volume – is a unique Open Access international journal which publishes articles in the fields of nanoscience, nanotechnology, nanobiotechnology, and single-molecule research, and has successfully published cutting-edge research results and reviews in these areas over the past three years (vols. 1–3). The importance of nanoscience and nanotechnology for future technology and health care is now well recognized, which is apparent from the ever-accelerating growth of interfaces among the aforementioned areas, applied research, and innovative technologies. Additional evidence that highlights the importance of basic research in these fields is the improved funding situation (despite the current economic climate), growth in research publications, human resources being dedicated to this area, and a terrific infrastructure. Recent developments along the interfaces between nanoscience, nanotechnology, and biology not only transcend the limits of traditional chemistry, physics, and biology but also demand all-inclusive information for education, research, and application. To meet this demand and to ensure that we are filling the growing need for an exchange of comprehensive information, our editorial team is striving to publish well-organized review articles in addition to original findings. In other words, the purpose of *Nano Reviews* is to organize and disseminate cutting-edge and high-quality research results in the above areas in a comprehensive fashion. In *Nano Reviews*, we gather new experimental and theoretical developments in nanoscience, nanotechnology, nanobiotechnology, and single-molecule research aspects in materials science, bioinformatics, bioconjugate chemistry, biophysics, biological chemistry, biotechnology, and medical and engineering sciences and offer readers worldwide Open Access to these publications. In this way, we offer high-quality reference materials for research and development, which will contribute to an investigation into the entire spectrum of nanoscience, nanotechnology, nanobiotechnology, and single-molecule research.

*Vasudevanpillai Biju* Editor-in-Chief

